# Nightmare Rescripting: Using Imagery Techniques to Treat Sleep Disturbances in Post-traumatic Stress Disorder

**DOI:** 10.3389/fpsyt.2022.866144

**Published:** 2022-04-04

**Authors:** Marzia Albanese, Marianna Liotti, Lucia Cornacchia, Francesco Mancini

**Affiliations:** ^1^School of Cognitive Psychotherapy, Rome, Italy; ^2^Crossing Dialogues Association, Rome, Italy; ^3^Department of Dynamic and Clinical Psychology, and Health Studies, Sapienza University of Rome, Rome, Italy; ^4^Department of Human Sciences, Guglielmo Marconi University, Rome, Italy

**Keywords:** trauma, nightmares, post-traumatic stress disorder, insomnia, imagery rescripting, imagery rehearsal therapy

## Abstract

Besides affecting 8% of the general population, nightmares are one of the most frequent symptoms of traumatized individuals. This can be a significant factor in the treatment of post-traumatic disorders; indeed, several studies demonstrated its strong predictive and prognostic value. Sleep disorders, nightmares in particular, could be very distressing for individuals and need targeted interventions, especially if they are associated with a PTSD diagnosis. To date, the best technique for the treatment of traumatic sleep disturbances seems to be Imagery Rehearsal Therapy (IRT), an empirically supported method. Through a review of the literature on this matter, this article aims to outline the incidence and consequences of nightmares in PTSD, illustrate how IRT could prove useful in their treatment, and investigate its clinical applications.

Post-traumatic stress disorder (PTSD) is a mental health condition, which in the fifth edition of the Diagnostic and Statistical Manual of Mental Disorders [DSM-5; (1)] has been included in a new category, “Trauma and Stressor Related Disorders.” PTSD is characterized by the appearance of a wide array of symptoms after experiencing “death, threatened death, actual or threatened serious injury, or actual or threatened sexual violence” [(1), p. 271], in the following ways: direct exposure to the event; witnessing the event; learning that a close one was exposed to a traumatic event; indirect exposure to details of the trauma.

PTSD diagnosis was added–not without many controversies–only in the third edition of the DSM [DSM-III; (2)], after noticing the development of post-traumatic symptoms among many veteran soldiers. However, it is possible to identify some descriptions ascribable to this disorder already at the beginning of the twentieth century, when many authors spoke of “war neurosis,” “soldier's heart,” and “shell shock” to describe the physio-psychological consequences of being exposed to war situations [for a historical overview, see (3)]. Shortly after the diagnosis of PTSD was introduced in the DSM, clinicians began to notice that there were other individuals–victims of sexual or physical abuse, for example–whose symptoms largely corresponded with those observed in soldiers. Today we know that the traumatic events that can give rise to PTSD are numerous and of various kinds. They produce lasting effects, which the DSM-5 describes as follows, dividing them into four clusters:

1) Re-experience of the traumatic event (intrusion symptoms) through distressing memories, dreams and nightmares, flashbacks, and dissociative reactions.
2) Avoidance of stimuli associated with the traumatic event.
3) Negative alterations in cognition and mood (e.g., amnesia, negative beliefs and expectations, distorted cognitions, feelings of detachment).
4) Marked alterations in arousal and reactivity (e.g., irritability, self-destructive behaviors, hypervigilance, difficulties in concentrating, sleep disturbances).

According to the DSM-5, in the United States PTSD affects ~5% of men and 10% of women (1). In Italy, epidemiological studies show that about 56.1% of the general population is exposed to at least one traumatic event (with an average of 4 traumatic events experienced during the lifespan); the risk of experiencing PTSD following exposure to a traumatic event(s) is assessed to be between 0.8 and 12.2% (4). These data highlight the significance of a better understanding of the complex symptoms that are often associated with PTSD to develop targeted and effective intervention techniques.

## Sleep Disturbances and Nightmares in PTSD

Within the heterogeneous post-traumatic symptomatology, sleep disorders seem to occupy a particularly prominent place (5–8). “O memory, mortal enemy of my rest!,” wrote Cervantes, enclosing–perhaps unconsciously–in these few words the struggle of those who, following a traumatic experience, continue to be haunted daily by the repetition of intrusive images, both during their wake and their sleep.

There are several studies [for a review, see (9)] that show how having experienced a traumatic event leads to difficulties in falling and/or staying asleep, as well as in having persistent and distressing nightmares. These consequences are part of a typical response to a traumatic event (10), so much that the DSM has always listed nightmares within the cluster B symptoms (re-experiencing), while a difficulty to falling asleep and/or staying asleep can be found among in Cluster D symptoms (arousal alterations).

Following the 1995 earthquake in Hanshin, Japan, Kat et al. (11) interviewed 143 people to study what effects the event had on them. The authors highlighted that sleep disturbances were the most common and frequently experienced symptom: 63% of the sample experienced it 3 weeks after the event, and 46% still 8 weeks later. Similarly, Goldstein et al. (12) and Kuch et al. (13) have highlighted the pervasiveness of these symptoms in trauma victims. Sleep disturbances were found in 97% of war soldiers and 95% of Holocaust survivors. Moreover, nightmares represented the major problem for both the former (94%) and the latter (83%).

Events such as the Oklahoma City attack and the World Trade Center attack have made it possible to study the dramatic impact of such incidents on the population involved, highlighting the essential link between trauma and sleep disorders. For example, more than 70% of Oklahoma City bombing survivors reported sleep disturbances 6 months after the event; ~50% of the analyzed sample also reported experiencing distressing nightmares (14). After the terrorist attack of September 11th, Schuster et al. (15) investigated the reaction of 560 American adults and children. Despite the different ages of the individuals who made up the sample, the authors found similar reactions among all subjects: 11% of adult individuals and 10% of children reported significant difficulties both in falling and staying asleep due to the presence of disturbing nightmares.

Another line of research (16–19) mainly focused on investigating the content of nightmares of people diagnosed with PTSD, showing interesting results. Indeed, nightmares seem to be not only and necessarily a reproduction of the particular kind of traumatic event experienced by the subject (19) but also a reflection of the intense emotions associated with it. Even after some time has passed (in which nightmares tend to “become chronic”), the nightmares of patients suffering from PTSD seem to contain general traumatic themes, such as threatening or deadly circumstances (18, 20). Investigating the reactions of a sample of Vietnam veterans diagnosed with PTSD, Esposito et al. (20) found that the dream content of these patients included not only combat scenarios set in the past but also other kinds of threatening scenarios set in the present. Furthermore, Dagan et al. (21) have found that PTSD patient's dreams are characterized by more violence and aggressivity than those of the general population.

In light of these findings, it seems crucial to understand how the presence and persistence of nightmares and sleep disturbances affect the overall clinical picture of PTSD, giving rise to secondary implications that require targeted interventions. Inman et al. (22) compared a sample of 35 Vietnam veterans diagnosed with PTSD with a non-clinical sample of 37 patients suffering from insomnia. Although there was no significant difference between the two groups in terms of the total amount of sleep (an average of about 2.7 h per night in both groups), individuals suffering from PTSD showed more sleep-related anxiety symptoms, including fear of falling asleep, fear of the dark, waking up with covers torn apart, yelling/shouting in their sleep, psychomotor agitation during sleep, waking up in a confused and disoriented state, and trying to stay awake for most of the night. Hefez et al. (23) have shown that noncombat subjects suffer from the same sleep disturbances found in Vietnam veterans.

In addition to offering an important starting point for considering how nightmares and sleep disturbances represent not only a distressing symptom but also an essential maintaining factor of PTSD–as commonly observed in clinical practice–, these results raise another relevant question: can sleep disturbances and nightmares be considered a prognostic and predictive factor of posttraumatic stress disorder? Harvey and Bryant (24) have examined the predictive power of every posttraumatic symptom. Their study, conducted on adult trauma survivors at 1 and 6 months after a traumatic incident, highlighted how individuals who were suffering from nightmares at 1 month after trauma presented a PTSD diagnosis 6 months later in 33% of the cases; those who were suffering from sleep disturbances at 1 month after trauma were diagnosed with PTSD in 72% of the cases. These findings suggest that the answer to our previous question is affirmative.

Koren et al. (25) have shown that sleep disturbances are a decisive prognostic factor in PTSD. The authors conducted a study on 102 adult patients who had survived motor vehicle accidents, administering them the *Mini Sleep Questionnaire* (26) 1 week after the traumatic incident and then 1, 3, 6, and 12 months later. Individuals who developed PTSD over time showed greater insomnia and sleepiness during the day than those who didn't develop it. Moreover, insomnia 1 month after the traumatic accident was not only predictive of PTSD later, but it also persisted until the end of assessment (i.e., 12 months after the trauma).

Recent studies (27–30) have observed a significant relationship between nightmares and suicidal behavior in patients with PTSD. The reason for this relationship is not yet completely understood. Littlewood et al. (31) proposed the association between nightmares and suicidal behaviors is due to the sense of defeat, entrapment, and despair inevitably experienced by those who continue to relive traumas in their dreams. Indeed, in their study, suicidal behaviors were higher in participants who experienced nightmares (62%), in comparison to those who did not (20%), regardless of comorbid insomnia and depression (31).

All the studies summarized above confirm the relevance of sleep disturbances and nightmares in PTSD and offer useful information for psychotherapeutic intervention. Indeed, it seems vital to find a specific intervention model for reducing the distress these symptoms cause. To date, the best technique for the treatment of traumatic sleep disturbances seems to be *Imagery Rehearsal Therapy* (IRT). In the following paragraphs, we will summarize how this approach was developed and why it is useful not only for reducing the distress caused by nightmares or other sleep disturbances but also for lessening the overall PTSD symptomatology.

## Imagery Rehearsal Therapy: A Specific Treatment for Nightmares

In light of the data reported in the previous paragraphs, it is important to develop a specific approach for treating sleep disorders–particularly nightmares–in patients with a PTSD diagnosis. Numerous authors with a cognitive-behavioral orientation have suggested using techniques that involve working with “mental images” (32–37). According to them, these techniques could represent the elective treatment for nightmares related to PTSD because they simultaneously allow the clinician to (a) have direct access to the content of the nightmare and to the emotions associated with it; (b) identify and modify negative cognitions related to the traumatic event; (c) reduce post-traumatic symptoms. Both Krakow et al. (38) and Davis and Wright (39) have identified, among the wide range of techniques for working with mental images, those that allow a more effective rescripting.

Imagery Rehearsal Therapy (IRT), a cognitive-behavioral procedure aimed at reducing the frequency and distressing impact of nightmares, was developed by Ian Marks in 1967 and subsequently perfected by Barry James Krakow and other psychotherapists interested in its use in the treatment of post-traumatic symptoms. This procedure, based on the work of Bootzin and Nicassio (40) and Howoritz (41), was developed within a theoretical framework that considers nightmares as a “learned” sleep disorder, which is, in turn, the result of distortions in the individual's mental images. From this perspective, nightmares are no longer considered solely as a post-traumatic symptom, thus treatable only through trauma-focused psychotherapy. Rather, they become a distinct phenomenon that, although originally connected with the trauma, tends to become chronic and to have a “life of its own,” thus becoming a maintaining factor of PTSD and aggravating its overall symptomatology.

The IRT procedure consists of several sessions of variable duration, frequency, and number, depending on the specificity of the clinical case. In any case, the intervention is divided as follows:

1) *Psychoeducation*. The clinician offers the patient–and family members, if needed–detailed information about dreams and their association with traumatic experiences. In this phase, the patient is also given instructions on proper sleep hygiene and dysfunctional behaviors that maintain insomnia.
2) *Learning*. The aim of this phase is to help the patient “learn” how to cope with nightmares through the acquisition of techniques for working on his mental images to develop pleasant ones. Among the techniques proposed by Krakow (42), which the clinician will have to select on the basis of the patient's particular preferences, are the use of color, shapes, and movement as “basic tools” for constructing positive mental images (*visual sense*); *daydreaming*, which, if done with awareness, can encourage the emergence of positive images; and *self-talk*, through which the patient associates a positive word with a pleasant image or story. All these techniques help the subject to “stock up” on positive images that they can later take with them during *imagery practice*. This practice, which is the focus of the second part of the intervention, helps patients construct a new “script” of their dreams. In this second phase, the therapist also guides patients to manage their disturbing mental images and assesses their ability to do so. Working with disturbing mental images can indeed lead patients to experience intrusive mental images related to the traumatic event, which can be overwhelming and provoke dissociative phenomena. Therefore, the patient is provided with grounding and self-regulation techniques. Before moving to the next phase, the therapist must assess the patient's ability to manage any distressing element that may emerge.
3) *Selecting the disturbing nightmare*. Once the patient has acquired the techniques for working on his mental images and has acquired the necessary skills for managing potential dissociative responses, the target nightmare for the intervention is selected. This is usually the most emotionally disturbing or the most frequent one (these two aspects often coincide). In some other cases, the patient is asked to start from a nightmare of lesser intensity so as to not feel overwhelmed.
4) *Re-evocation of the selected nightmare*. The patient is asked to write down the chosen nightmare and is encouraged to note all its details.
5) *Nightmare rescripting*. Using the skills learned during phase two, the patient can now make any changes they deem necessary on his nightmare. In this stage, which is at the heart of the IRT procedure, patients are offered the possibility of transforming their nightmare into a positive one–changing its theme, plot, ending, or any other part that they believe could help them in the rescripting. Interestingly, a study by Harb et al. (43) has shown that the majority of individuals (58%) created alternative endings. Others chose to insert new positive images without changing the ending (23%); a smaller percentage preferred to transform the threatening elements of the nightmare into less distressing images (13%). Finally, 10% of subjects decided to insert “reminders” (e.g., objects) that would help them be aware that they were simply dreaming, while 8% used distancing techniques (8%).
6) *Rehearsal*. The patient is invited to mentally rehears the rescripting of the selected nightmare for at least 10–20 min a day (preferably before going to sleep) until they obtain a significant reduction in the frequency of the nightmare. For this to happen, they should repeat only the new script without recalling the original nightmare (42). According to the rationale of the technique, the constant repetition of the new images created by the patient leads to a modification of the contents of the nightmare.

IRT works by progressively “inhibiting” the original nightmare, which is replaced with positive elements that can overcome the disturbing power of the unwanted contents of the dream. This leads to a change in the negative cognitions and feelings associated with the previous contents of the nightmare. We believe that this is the factor that makes the IRT such a helpful tool since it is plausible that such changes will be extended to other domains of functioning.

There are currently several variants of IRT, such as the Imagery Rescripting and Exposure Therapy (IRET) (44) and the Exposure, Relaxation, and Rescripting Therapy (ERRT) (45). These share with IRT not only the same rationale but also almost all the procedures listed above, even if with some modifications: IRET also uses relaxation techniques (e.g., progressive muscle relaxation) in the learning phase, while ERRT introduces “nightmare exposure” techniques, which are similar to imagery practice.

Currently, according to the American Academy of Sleep Medicine (AASM), IRT represents the treatment of choice (Level A) for PTSD-associated nightmares and nightmare disorder (5, 46). The present narrative review aims at describing and discussing the studies that analyzed the efficacy of IRT in the treatment of nightmares in post-traumatic populations (e.g., veterans, victims of sexual abuse). To this aim, a literature search was conducted using the following databases: Scopus, PsycINFO, PsycARTICLES, PubMed, Web of Science, and Google Scholar. The keywords for the search were: “nightmare,” “nightmare disorder,” “PTSD,” “imagery rehearsal therapy,” “nightmare rescripting,” “sleep disturbance,” “sleep disorder,” “imagery rescripting,” used in different combinations. Studies investigating the application of imaginative techniques to other psychopathologies (e.g., anxiety disorders) were not included.

## Clinical Applications With Adult Patients

### War Veterans

In the adult clinical population, IRT seems to be particularly effective in reducing nightmares in war veterans who have developed PTSD (43, 44, 47, 48). To meet the specific needs of the veteran population, Long et al. (44) applied the IRET, a variant of the initial model. The authors investigated the effectiveness of the rescripting treatment, carried out over six sessions, on a group of 37 US veterans with PTSD and chronic post-traumatic nightmares (present for 10 years). Of the 33 individuals who completed the treatment, 15.2% reported that they had not subsequently suffered from distressing nightmares. 30.3% reported a significant increase in the amount of time dedicated to sleep, which became 6 h or more per night. Besides, 30 of the 33 patients (90.9%) reported general, albeit moderate, improvements in their sleep disturbances.

Other studies on the use and effectiveness of IRT and its variations with war veterans have focused on the peculiar qualities of their nightmares and how these could be modified by rescripting, analyzing the correlation between these elements and the positive outcome of treatment. Harb et al. (43) examined the characteristics of the distressing nightmares of 48 US veterans suffering from PTSD. The aim of the research was to outline if and how specific characteristics of the nightmares were associated with the treatment outcome. Compared to other survivors of traumatic events, war veterans presented significantly more “replicative” nightmares, characterized by reproductions of the original traumatic event or parts of it–such as bodily sensations, emotions, smells, or sounds. About half of the veteran's nightmares considered in the study (between 21 and 60%) contained scenarios, individuals, or objects characteristic of the original traumatic event(s). This study also highlighted that the vividness and intensity of the olfactory sensations in patient's nightmares were inversely related to their response to IRT treatment (patients with vivid and intense olfactory experiences tended to have a poorer response). Compared to other forms of sensory memory, smell is more closely connected to affects. The processing of olfactory stimuli involves the activation of primitive brain structures also implicated in fear and survival responses. Instead, a factor correlated with the success of the IRT was the patient's ability to incorporate a resolution of the central theme of the replicative nightmare in their rescripting, eliminating its most violent details. The most effective strategies in modifying the content of the nightmare and in resolving the distress it produced in PTSD patients were the conception of alternative endings, the insertion of resolving elements during violent scenes, the transformation of threatening objects such as weapons into harmless objects, and/or the use of distancing techniques from the source of threat.

These results appear consistent with the literature on the cognitive-behavioral treatment of PTSD: the IRT seems to represent a form of cognitive restructuring of the meaning of the traumatic memory reproduced in the patient's nightmares. Long et al. (44) showed that the central themes of war veteran's nightmares revolved mainly around impotence (a feeling experienced by 90% of subjects and present in 27.1% of targeted nightmares), fear of death (experienced by 85% of subjects and present in 41.7% of targeted nightmares), and a feeling of lack of control and self-efficacy (present in 27.1% of targeted nightmares). The results of this study confirm that, during the rescripting phase, focusing on the central theme reported by the patient is associated with a positive treatment outcome. Indeed, the main themes present in the patient's new dreams, as well as the most effective elements in reducing their suffering, were related to the presence of positive, optimistic feelings (37.5%), a sense of security and peace (27.5%) and a feeling of greater self-efficacy (25%).

Moreover, the positive results of IRT seem to be long-lasting. Moore and Krakow (48) conducted a study on 7 soldiers who, after returning from their mission in Iraq, had developed distressing nightmares and sleep disturbances. At the end of the IRT treatment, they reported a significant reduction in the frequency of nightmares: at the 1 month follow-up, the mean number of nightmares diminished by 44%. Moreover, 64% of soldiers reported a significant relief regarding not only their nightmares but also their PTSD symptoms and insomnia, which diminished, respectively, by 41 and 34%.

The positive effects of IRT seem to last after the end of treatment, as confirmed by a study by Lu et al. (47). The study focused on 17 war veterans with a PTSD diagnosis, who were invited to participate in an IRT group training, with 6 weekly sessions of 90 min. Initially, contrary to what was expected, no significant results were found. However, during follow-up evaluations (at 3 and 6 months), the patients reported that the frequency and number of their nightmares were significantly reduced, that such nightmares were less distressing and that even their fear of being asleep considerably diminished. While no improvements in sleep quality or depression scales were found, a marked decrease in post-traumatic symptoms was observed.

These results suggest that the specific positive effects of IRT treatment may appear even after some time from its conclusion. Consistently with the results of other efficacy studies on IRT (49), the study by Lu et al. (47) shows how IRT can be effective in the treatment of distressing nightmares on veterans suffering from lasting PTSD symptoms, even after they have concluded other forms of treatment [see also (50)].

In another study, Ulmer et al. (51) treated 22 veterans with PTSD with a combination of CBT and IRT; the intervention consisted of 6 bi-weekly sessions for 12 weeks. The combined CBT/IRT intervention produced significantly greater improvements in nightmare frequency compared to usual care (51).

The limitations of the studies reviewed here are mainly of two types: the first concerns the non-representativeness of the sample, as this is often too small or restricted to a specific age range; the second concerns the fact that female subjects are excluded from these studies. Moreover, it would be interesting to extend the follow-up period to further support the long-term efficacy of IRT with war veterans.

### Victims of Sexual Abuse

In addition to war veterans, IRT is also effective in reducing the distress caused by nightmares in subjects who have suffered sexual abuse, an experience that often leads to the development of PTSD. Davis and Wright (45) reported that sleep disturbances and nightmares are extremely frequent symptoms in victims of abuse; they can act as a maintenance factor for post-traumatic stress symptoms and/or lead to the development of depressive symptoms. Cognitive Behavioral Therapy (CBT), however, often does not involve interventions aimed at directly addressing this type of problem. According to Belleville et al. (52), although CBT typically leads to spontaneous improvement in sleep-related difficulties, these often resurface about 6 months after treatment ends. Therefore, the authors have highlighted the need to develop guidelines and standardized procedures for the treatment of sleep disorders in subjects suffering from PTSD. Thus, they have investigated through a randomized controlled trial the effects of IRT on sleep disturbances (e.g., insomnia, nightmares), as well as other PTSD symptoms, general functioning, and quality of life, comparing this treatment to the application of CBT alone. 42 adult subjects with a history of sexual abuse and a diagnosis of PTSD were thus recruited and then randomly assigned to the experimental condition (IRT+CBT) or the control condition (CBT only). Before starting CBT, the women assigned to the experimental group participated in 5 weekly sessions in which IRT was applied to their most disturbing nightmare. Subsequently, all subjects received 15 CBT sessions. The results of the study showed that even if at the end of treatment both groups showed a significant–and similar–decrease in PTSD symptoms, associated with an improvement in general functioning and quality of life, the group receiving the IRT showed a more substantial improvement in sleep quality and a greater decrease in the frequency of nightmares. The authors, therefore, concluded that IRT appears to be a valid technique for those patients who cannot benefit from long-term therapies or for whom sleep disorders represent a primary element to be addressed during treatment (52).

Similar results are also found in other studies, albeit less recent. Among these, there is only one other randomized controlled trial, conducted by Krakow et al. (53) on a sample of 114 adult women victims of sexual abuse and suffering from insomnia and other PTSD symptoms. The women were randomly assigned to an experimental group (*cognitive imagery treatment*) or a control group (*no imagery intervention, continuation of standard treatment*). The experimental group received three weekly group sessions: the first two lasting 3 h each, the last lasting 1 h. During the second session, the participants were asked to write down their most disturbing nightmare, modifying it “however they wanted”–according to the model of Neidhart et al. (54). Soon after, the women were asked to use imagery rescripting to mentally visualize their new dream and to do so multiple times. At the end of the study, the women assigned to the cognitive imagery treatment group showed significantly fewer nightmares per week and better sleep quality than the control group; besides, their other post-traumatic stress symptoms lessened significantly more. The experimental group maintained these improvements even at follow-up (at 3 and 6 months), while the control group continued to show slight or no developments. Furthermore, the differences between the two groups were not mediated by variables such as the specific psychotherapeutic or pharmacological treatment previously received by each woman. The authors conclude that IRT is a technique that can be beneficial for victims of abuse, thanks to its many positive effects and its brevity. They recommend the use of IRT in the treatment of patients with post-traumatic symptoms or, more generally, patients suffering from sleep disorders. They also highlight the usefulness of IRT in cases where subjects are resistant to the idea of pharmacological treatment, while underlining the need for further studies to better understand the factors and mechanisms that make this technique effective in reducing traumatized subject's sleep difficulties and the disturbing emotional charge associated with their dreams (53).

Similar considerations can be found in a study by Germain et al. (33), which aim was to investigate if the increased sense of mastery may be among the main factors that explain the effectiveness of IRT in the treatment of post-traumatic nightmares. The authors applied the same procedure previously used by Krakow et al. (53). In this case, it was used during the treatment of 44 sexually abused women suffering from nightmares, insomnia, and other PTSD symptoms. The results showed that not only the new dreams produced with IRT were characterized by fewer negative elements, but that the subjects developed a greater sense of mastery regarding the negative elements of their nightmare, thus feeling more confident of being able to cope with them. According to the authors, this increased sense of mastery could prove useful in reducing not only sleep disorders but also other PTSD symptoms, such as intrusive memories, flashbacks, or the avoidance of stimuli and situations associated with trauma. In addition to the fact that it requires a relatively limited time to produce the first beneficial effects on traumatized subjects, one of the advantages of IRT is its simplicity, which makes it usable even in cases in which other interventions may prove less effective, for example with individuals with learning and intellectual disabilities.

Kroese and Thomas (55) conducted a case study on two sexually abused women with intellectual disabilities, showing that the rescripting of the most disturbing images characterizing their recurring nightmares (which were representations of the trauma they experienced) proved to be useful to modify the pathogenic beliefs relating to the victim's sense of helplessness. IRT also increased their sense of control and mastery over the traumatic situation represented in the dream–effects that, in line with previous research, seemed to generalize to other everyday situations.

Davis and Wright (45) have highlighted the usefulness of imagery rescripting in reducing PTSD symptoms not only in the adult population but also in adolescents. In their study, the participants (1 male and 3 females, all victims of sexual abuse) received a modified version of the technique, the ERRT. The participants were offered three sessions in total, lasting 2 h each. The results of the study once again confirm the usefulness of this technique: indeed, the rescripting of mental images characterizing the nightmare not only reduces sleep disorders related to PTSD but also has beneficial effects on post-traumatic symptomatology as a whole. However, the authors stress the need for further studies to shed light on the mechanisms underlying the effectiveness of the technique. The fact that IRT has also proved effective in a sample of adolescents, though, leads us to consider another population on which the use of nightmare rescripting seems to offer numerous benefits: that of developmental age.

## Clinical Applications With Children and Adolescents

In a chapter of the volume “Innovations and Advances in Cognitive Behavior Therapy,” Encel and Dohnt (56) highlight how, although nightmares and sleep disturbances are among the symptoms most frequently reported by children with PTSD, these rarely represent a direct target of cognitive-behavioral oriented therapies. Furthermore, post-traumatic nightmares in children often prove to be persistent and cause considerable distress: nightmares can damage the daily functioning of the child and aggravate their clinical picture (56). After highlighting the need for techniques specifically aimed at the treatment of nightmares in children, Encel and Dohnt suggest some changes to the IRT procedure that could make it more suitable for developmental age and underline the need for controlled studies to verify its efficacy in the treatment of sleep disorders in childhood.

Although thirteen years have gone by, such studies remain, to our knowledge, quite limited. Yet those conducted so far all suggest promising results. St-Onge et al. (57) conducted a randomized controlled trial to see if IRT could be useful for the treatment of prepubertal children suffering from frequent and chronic nightmares. They assigned 20 children (eleven boys and nine girls, aged 9 to 11) to an experimental group (*IRT*) or a control group (*waiting list*). After an initial psychoeducational session about sleep and nightmares, which was offered to all the subjects of the study, about 4 weeks later the children assigned to the experimental group participated in a second session, in which they were explained how to autonomously apply the IRT for the next 8 weeks. The use of IRT led to a significant reduction in the frequency of nightmares in the experimental group. This improvement was also maintained at the 9-month follow-up, thus confirming the usefulness and suitability of IRT with children. Similarly, Simard and Nielsen (58) found that administering a single session of IRT to a group of children (aged 6 to 11) with sleep disturbances, but without a PTSD diagnosis, led to a reduction in the distress caused by nightmares and to a decrease of other anxious and depressive symptoms.

The only study investigating the efficacy of imagery rescripting in a sample of children suffering not only from frequent and/or chronic nightmares but also from PTSD was conducted by Fernandez et al. (59), who have applied the ERRT on two girls of eight and eleven years of age respectively. Again, the results seem promising: the authors report an increase in sleep quality and a decrease in the frequency of nightmares in both girls, as well as fewer parental observations of behavioral problems in their children.

In conclusion, although further studies are needed (preferably randomized controlled trials with sufficiently large samples), it appears that IRT may be a useful technique to integrate into cognitive-behavioral treatment for children with nightmares and sleep disorders. Indeed, these symptoms often represent a problem of great importance during developmental age, especially with respect to the presence of a PTSD diagnosis; to date, however, there is a lack of proven techniques for their treatment.

## Discussion

Nightmares are a prominent symptom of PTSD and other stress-related disorders (5–8), and they often seem to resist classic trauma-focused psychotherapeutic interventions. Moreover, even if reduced, they tend to reappear in the follow-up assessments after several months (60, 61). Even when PTSD resolves, nightmares can persist (5, 46).

But what makes this symptom so central and “resistant” to treatment, and therefore so important to treat? Clinical practice offers interesting and useful observations on this matter, which found confirmation in the literature. Patients who suffer from frequent nightmares experience greater levels of insomnia; their resulting fatigue seems to contribute to the experience of dissociative phenomena, which are both more frequent and more intense in individuals suffering from nightmares and sleep deprivation. Several studies (62–65) have highlighted a correlation between dissociative symptoms and sleep disturbances: while insomnia induces dissociative symptoms (66), sleep improvement reduces them (63).

Furthermore, in clinical practice, we often observe that the presence of nightmares triggers vicious circles that not only act as a maintaining factor of PTSD but also, in more serious cases, aggravate its symptoms. This happens because the constant presence of nightmares leads patients to experience strong anticipatory anxiety before going to sleep. It is not uncommon to hear patients tell us of their abuse of alcohol or other drugs in the attempt of self-medicating, with the illusion that by using these substances, they will be able to cope with their anxiety and go to sleep “undisturbed.”

Nightmares contribute to the maintenance of PTSD symptoms also because they sometimes prevent its treatment. Indeed, the insomnia experienced by traumatized individuals due to trauma-related nightmares and subsequent recurrent awakenings often leads them to have difficulties concentrating during the day. This can seriously inhibit their ability to carry out a wide number of activities–including psychotherapy. During the sessions, a patient suffering from sleep disorders may have difficulties in listening to the psychotherapist and actively participate in therapy precisely because of his/her fatigue. The fact that the presence of nightmares tends to exacerbate other symptoms of PTSD, hindering the success of psychotherapy, finds numerous empirical validations. Several studies (39, 67, 68) have highlighted this phenomenon, also suggesting that nightmares contribute to increasing the subject's general levels of psychological, above and beyond the severity of PTSD itself.

Finally, in clinical practice we often observe that nightmares play a central role in maintaining and strengthening the traumatized subject's pathogenic beliefs. Nightmares, especially if they are chronic, are indeed often experienced by such individuals as an uncontrollable and intrusive phenomenon, leading patients to tell themselves things like: “I am broken,” “I have no hope of healing,” “Things will never go back to the way they used to be,” “This thing will haunt me forever,” “I am no longer in control,” “I am helpless,” and so on.

We believe that this is the element that makes a specific model of treatment for nightmares effective and necessary: a specific intervention can indeed modify the patient's pathogenic beliefs related to trauma and, subsequently, foster a reduction of their PTSD symptoms and of their levels of psychological maladjustment (35). As we've seen, the reduction of PTSD symptoms correlates with the decrease in the perception of impotence gained through the application of techniques such as IRT (44).

These observations are also confirmed by recent studies (69, 70). Rosseau and Belleville (70) systematically reviewed the supposed mechanisms of action of existing nightmare treatments, revealing that an increased sense of mastery was the most often cited hypothesis to explain the efficacy of nightmare psychotherapies. Similarly, Kunze et al. (69) found that enhanced mastery (or self-efficacy) mediates the therapeutic efficacy of imagery rescripting (IR).

In summary, clinical observations and empirical data indicate that a treatment specifically aimed at reducing nightmares in trauma survivors is vital, since nightmares contribute to the development and maintenance of post-traumatic symptoms (71), while their treatment favors the resolution of PTSD.

## Conclusions

Among the methods proposed for the treatment of nightmares in patients with PTSD, those involving imagery rescripting seem to be the most effective (32–39, 72). These techniques allow direct access to the distressing contents and emotions of nightmares without being overwhelming for patients, and they allow clinicians to quickly identify and modify trauma-related negative beliefs.

Rescripting-based therapy is generally thought to change the affective properties of a nightmare by altering its intrinsic meaning and by influencing the patient's ability to control distressing nightmare images (69). In nightmare disorder, this is very important because dreams are primarily hallmarked by a lack of self-efficacy, powerlessness and uncontrollability. IR offers means to help patients express their unmet needs and inhibited responses (73).

Today, the treatment of choice for working on nightmares is Imagery Rehearsal Therapy (IRT). This method involves a psychoeducation phase, as well as the implementation of exposure and imagery rescripting techniques, and is effective in the treatment of both adults and children, and adolescents.

Since it shares the same theoretical principles of Imagery Rescripting (IwR) (73–75), which has recently been used for working on dream scenarios, it is important to conduct further studies to evaluate its efficacy in the specific treatment of nightmares on different clinical samples. This could lead to the development of an integrated intervention that may allow clinicians to intervene effectively even in cases in which there are linguistic communication barriers, as often happens when working with migrants, asylum seekers, refugees, and victims of torture, who need an intervention that can take these difficulties into account.

## Author Contributions

MA contributed to concept, design of the review, and wrote the first draft of the manuscript. ML, LC, and FM contributed to search for scientific papers. MA, ML, and LC wrote sections of the manuscript. FM contributed to manuscript revision, read, and approved the submitted version. All authors contributed to the article and approved the submitted version.

## Conflict of Interest

The authors declare that the research was conducted in the absence of any commercial or financial relationships that could be construed as a potential conflict of interest. The handling editor declared a shared affiliation with one of the authors ML at time of review.

## Publisher's Note

All claims expressed in this article are solely those of the authors and do not necessarily represent those of their affiliated organizations, or those of the publisher, the editors and the reviewers. Any product that may be evaluated in this article, or claim that may be made by its manufacturer, is not guaranteed or endorsed by the publisher.
